# A literature review of dispersal pathways of *Aedes albopictus* across different spatial scales: implications for vector surveillance

**DOI:** 10.1186/s13071-022-05413-5

**Published:** 2022-08-27

**Authors:** Tom Swan, Tanya L. Russell, Kyran M. Staunton, Matt A. Field, Scott A. Ritchie, Thomas R. Burkot

**Affiliations:** 1 College of Public Health, Medical and Veterinary Sciences, James Cook University, Cairns, Australia; 2 Australian Institute of Tropical Health and Medicine, James Cook University, Cairns, Australia

**Keywords:** *Aedes albopictus*, Dispersal, Dispersal pathways, Spatial scales, Vector surveillance, Citizen science, Genomics

## Abstract

**Background:**

*Aedes albopictus* is a highly invasive species and an important vector of dengue and chikungunya viruses. Indigenous to Southeast Asia, *Ae. albopictus* has successfully invaded every inhabited continent, except Antarctica, in the past 80 years. Vector surveillance and control at points of entry (PoE) is the most critical front line of defence against the introduction of *Ae. albopictus* to new areas. Identifying the pathways by which *Ae. albopictus* are introduced is the key to implementing effective vector surveillance to rapidly detect introductions and to eliminate them.

**Methods:**

A literature review was conducted to identify studies and data sources reporting the known and suspected dispersal pathways of human-mediated *Ae. albopictus* dispersal between 1940–2020. Studies and data sources reporting the first introduction of *Ae. albopictus* in a new country were selected for data extraction and analyses.

**Results:**

Between 1940–2020, *Ae. albopictus* was reported via various dispersal pathways into 86 new countries. Two main dispersal pathways were identified: (1) at global and continental spatial scales, maritime sea transport was the main dispersal pathway for *Ae. albopictus* into new countries in the middle to late 20th Century, with ships carrying used tyres of particular importance during the 1980s and 1990s, and (2) at continental and national spatial scales, the passive transportation of *Ae. albopictus* in ground vehicles and to a lesser extent the trade of used tyres and maritime sea transport appear to be the major drivers of *Ae. albopictus* dispersal into new countries, especially in Europe. Finally, the dispersal pathways for the introduction and spread of *Ae. albopictus* in numerous countries remains unknown, especially from the 1990s onwards.

**Conclusions:**

This review identified the main known and suspected dispersal pathways of human-mediated *Ae. albopictus* dispersal leading to the first introduction of *Ae. albopictus* into new countries and highlighted gaps in our understanding of *Ae. albopictus* dispersal pathways. Relevant advances in vector surveillance and genomic tracking techniques are presented and discussed in the context of improving vector surveillance.

**Graphical Abstract:**

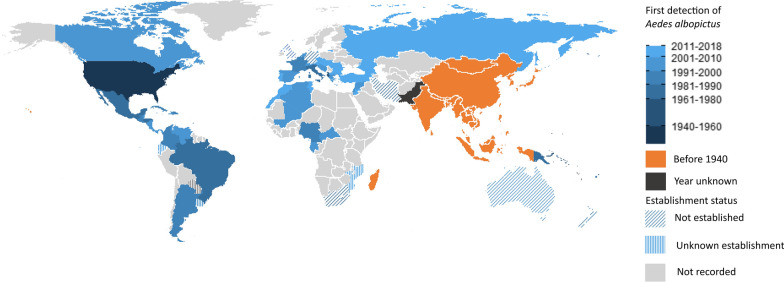

**Supplementary Information:**

The online version contains supplementary material available at 10.1186/s13071-022-05413-5.

## Background

**Fig. 1:**
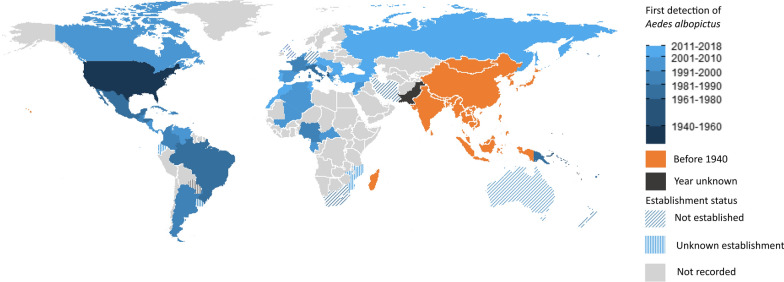
*Aedes albopictus* distribution range. Map indicates the year of first detections (interceptions and vector surveillance of *Ae. albopictus*) by country and whether established populations were formed (full colour). ‘Before 1940’ was based on published literature documenting the presence of *Ae. albopictus* populations in these countries before 1940. Establishment status was defined as persistent spatial and temporal published records. ‘Not established’ was defined as *Ae. albopictus* populations recorded sporadically after an incursion. In Australia, populations are only recorded in the Torres Strait, with no established populations on the Australian mainland. 'Unknown establishment’ was defined as no published records regarding its establishment after detections were made. ‘Not recorded’ was defined as no records of *Ae. albopictus* have been recorded for this country

*Aedes albopictus* is a highly invasive species [1]. Indigenous to Southeast Asia, *Ae. albopictus* has successfully invaded every inhabited continent, except Antarctica, in the past 80 years [2, 3] (Fig. 1). The invasion of new territories by *Ae. albopictus*, being dispersal occuring at broad spatial scales such as between continents (global), within continents (continental) and large distances within countries (national), usually occurs via passive dispersal. This form of dispersal is almost exclusively human-mediated and is believed to be driven by various dispersal pathways such as maritime transport, ground vehicles and the trade of both used tyres and lucky bamboo [4–6]. Range expansion in newly invaded areas may be facilitated by either passive or active dispersal. Active dispersal is defined as movement by mosquito flight and is generally highly localised with dispersal events usually limited to < 400 m [7–10].

Genetic evidence suggests that the *Ae. albopictus* worldwide invasion is strongly associated with human-mediated transportation (passive dispersal) [11]. As such, vector surveillance and control at points of entry (PoE) is the most critical front line of defence against the introduction of *Ae. albopictus* to new areas [12, 13]. Identifying the pathways by which *Ae. albopictus* are introduced is the key to implementing effective vector surveillance to rapidly detect introductions and to eliminate them [14]. Such knowledge could increase surveillance of common dispersal pathways including at PoE where *Ae. albopictus* are regularly intercepted [12, 15].

The main objective of this review is to examine the known and suspected human-mediated dispersal pathways of *Ae. albopictus* from 1940 to 2020. Second, techniques to determine *Ae. albopictus* dispersal across different spatial scales will be discussed and the implications for vector surveillance highlighted.

## Review methods

### Literature search and eligibility criteria

To investigate the dispersal pathways of *Ae. albopictus* into a country, published studies, reports, conference proceedings, grey literature and data sources investigating the human-mediated passive dispersal of *Aedes albopictus* were searched in Scopus, Web of Science and Google Scholar databases between November 2020–February 2021, using the following search terms “*Aedes albopictus*” OR “Asian Tiger Mosquito” AND “dispersal” OR “detection” OR “invasion” OR “Coloni*” OR “differentiation” OR “genetics” OR “surveillance” OR “movement” OR “long-range dispersal” OR “incurs*” OR “citizen*”. Reviews found in the initial search were also used to locate other papers relevant to the review question. In addition, the reference list of published studies of screened articles was searched for additional articles which were not included in the databases and of relevance to the review question. Search results from these databases were downloaded and Mendeley Desktop (v. 1.19.8) was used to remove duplicates. Inclusion criterion was publications reporting the first introduction of *Ae. albopictus* in a new country, with these publications selected for data extraction and analyses. Exclusion criteria were limited to non-English publications.

### Data collection process

To reduce selection bias, a standard data collection protocol was established to extract all relevant information for analysis. Authors, recorded dispersal pathway (if known), spatial scale (if known), time period of detection, year of first detection, recipient country (if known), donor country (if known), lifestage detected, trap used for detection (if reported), whether *Ae. albopictus* was detected at the PoE and the establishment status (determined in 2020 from information derived from agencies, organisations, reports and published scientific articles; Additional File 1: Table S1).

## Results

### Introduction of *Ae. albopictus* to new countries

**Fig. 2:**
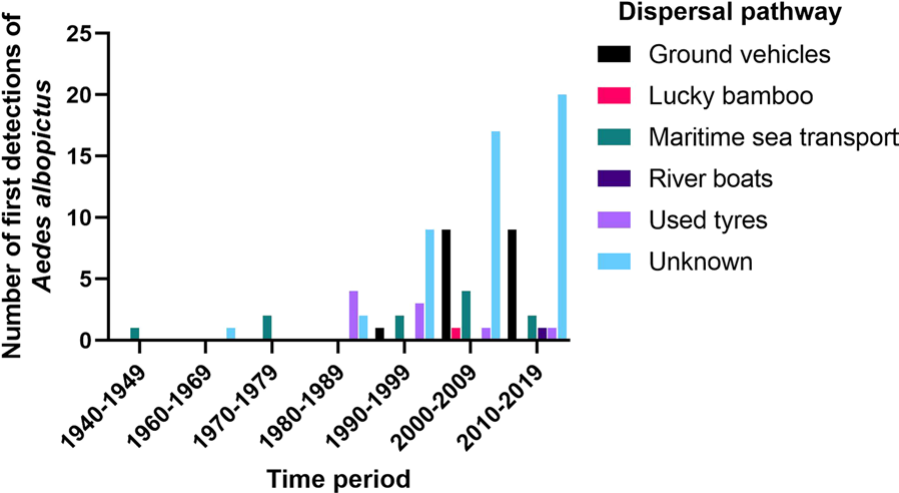
Number of first detections (interceptions and vector surveillance) of *Ae. albopictus* in a country by known and suspected dispersal pathways for the period 1940–2020. Publications reporting the first detection of *Ae. albopictus* in a new country were selected (Additional File 1: Table S1). "Unknown" dispersal pathway is defined from published scientific articles with insufficient evidence to prove or suspect otherwise

**Fig. 3:**
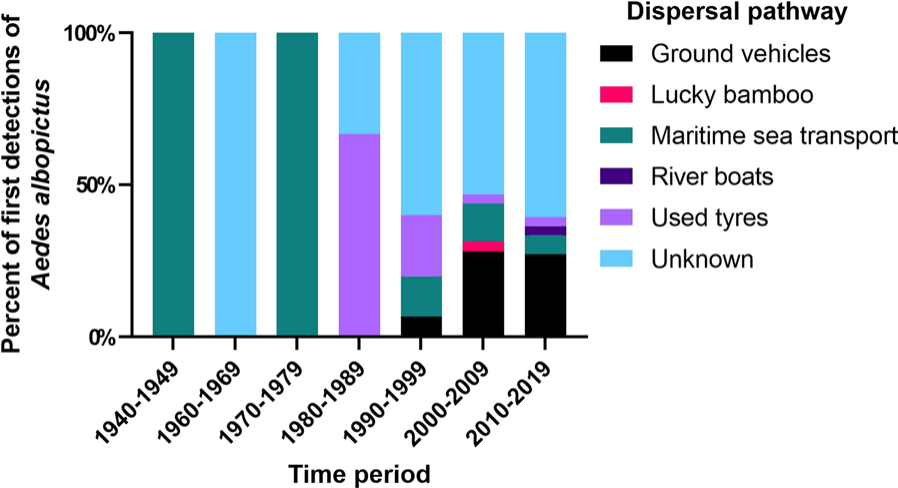
Percent of first detections (interceptions and vector surveillance) of *Ae. albopictus* in a country by known and suspected dispersal pathways for the period 1940–2020. Publications reporting the first detection of *Ae. albopictus* in a new country were selected (Additional File 1: Table S1). "Unknown" dispersal pathway is defined from published scientific articles with insufficient evidence to prove or suspect otherwise

For the period 1940–2020, *Ae. albopictus* was reported in 86 countries for the first time (Fig. 1, Additional File 1: Table S1). Maritime sea transport is the oldest documented dispersal pathway and remains an important pathway for the introduction of *Ae. albopictus* into new countries over time [2] (Figs. 2, 3). The transportation of used tyres is the second oldest dispersal pathway and between 1980–1999 presented one of the greatest risks for importation of *Ae. albopictus* worldwide (Figs. 2, 3). However, introductions of *Ae. albopictus* to new countries from this pathway has decreased with time, and in the last 2 decades, transportation of *Ae. albopictus* in ground vehicles was the main dispersal pathway for *Ae. albopictus* into new countries in Europe [6, 16–21] (Figs. 2, 3). The trade of lucky bamboo (*Dracaena* species) containing *Ae. albopictus* eggs has been recorded occasionally [22–24], but presents a lesser risk compared with the other known dispersal pathways (Figs. 2, 3). The passive transportation of *Ae. albopictus* by river boat has been recorded once [25] and like the trade of lucky bamboo presents a lesser risk compared with the other known dispersal pathways. This next section will discuss each documented dispersal pathway for the first introduction of *Ae. albopictus* in a new country, worldwide.

### Passive transportation by maritime sea transport

This dispersal pathway is typically characterised by the unintentional transportation of container habitats with eggs or immatures and the passive dispersal of *Ae. albopictus* adults on maritime sea vessels. This dispersal pathway functions over various spatial scales and is considered a major driver of *Ae. albopictus* dispersal to new countries with seaports.

In Australasia and countries in the Pacific, *Ae. albopictus* was discovered in Guam in 1944 [26]; its dispersal was most likely linked to the human movement of goods by ships to Guam during World War II. By the early 1970s *Ae. albopictus* was detected in northern Papua New Guinea (PNG) [27], Solomon Islands in 1979 [28] and Fiji in 1989 [29] with establishment of populations in the Solomon Islands and Fiji likely from PNG via shipping [30]. In 2005, *Aedes albopictus* was first detected in the Torres Strait Islands, Australia [31], introduced most likely from Indonesian fishing vessels [32]. Vector surveillance and control has prevented *Ae. albopictus* from establishing onto the Australian mainland [33, 34], despite detections at PoE throughout Australia [35–38].

In Europe, passive transportation of eggs, immatures and adult *Ae. albopictus* by maritime sea transport is considered a major driving contributor to *Ae. albopictus* introduction and establishment in the Mediterranean islands. Using this dispersal pathway, *Ae. albopictus* became established in Corsica in 2002 [39], the Greek Islands in 2003 [40], Malta in 2009 [41], Ibiza in 2014 [42] and the Tyrrhenian islands in 2016 [43].

### Trade of used tyres

The extensive global trade of used tyres, containing desiccation-resistant mosquito eggs, is historically one of the greatest risks for importation and dispersal of *Ae. albopictus* worldwide [2, 44] (Additional File 1: Table S1). Like maritime sea transport, dispersal via the used tyre trade operates over various spatial scales, has been linked with the initial *Ae. albopictus* invasion into various countries and is also likely a major driver for range expansion within countries.

In North America, *Ae. albopictus* immatures and adults were first detected in 1946 [45] in shipments of used aerial and military vehicle tyres returning to the port of Los Angeles via cargo ships from the Pacific following World War II. One shipment recorded larvae and adults of *Ae. albopictus* transported from Batangas, Philippines. To prevent the dispersal of *Ae. albopictus* from the port, infested tyres were sprayed with 5% DDT in kerosene and aerosol insecticides were applied to ship holds and rail cars [45]. No established populations of *Ae. albopictus* were recorded at this time and it was not until 1985 that populations of *Ae. albopictus* became established in Texas, probably transported from Japan to Texas in used tyres in 1985 [46, 47]. Dispersal from Texas is considered the origin for the rapid and widespread dispersal of this species in North America by various dispersal pathways across both continental and national spatial scales. In the USA, following the detection of *Ae. albopictus* in Texas in 1985, subsequent dispersal of tyres via vehicles on the interstate highway system was suggested as contributing to the rapid spread of this species throughout the country [48–50]. Given the preference of *Ae. albopictus* to use tyres for oviposition [1] and the widespread movement of tyres for retreading, recycling or other purposes in the USA [50], it has been suggested that humans greatly aided the dispersal of this species in this way [1]. As of 2017, *Ae. albopictus* was reported from 1368 counties in 40 states in the USA [51].

*Aedes albopictus* was first detected in Europe in Albania in 1979, when *Ae. albopictus* immatures were discovered in used tyres at a number of widely separated locations throughout the country [52]. *Aedes albopictus* were found in used tyres at the port city of Durres, suggesting that the China to Albania used tyre trade route transported via cargo ship was the likely source of infestations seen across the country [52]. Further infestations in Europe were not detected until *Ae. albopictus* immatures were found in imported used tyres in Italy in 1990 and later in France in 1999, with used tyres transported via cargo ships from the USA suggested as the origin of the mosquitoes [53–55]. The used tyre trade is considered the greatest risk of importation of *Ae. albopictus* at global and continental spatial scales into Europe [6]. *Aedes albopictus* is now widely spread throughout Europe and established in over 15 European countries (Fig. 1) [18, 56–58].

In Central and South America, *Ae. albopictus* was contemporaneously detected in the southeastern states of Rio de Janeiro and Minas Gerais, Brazil, in 1986 [59, 60], with dispersal throughout most of Brazil in succeeding decades [61]. The importation of used tyres via cargo ship containing *Ae. albopictus* immatures, of unknown origin, seems to have introduced *Ae. albopictus* into Brazil [39]. Following the invasion of *Ae. albopictus* in Brazil, *Ae. albopictus* surveillance programmes commenced in surrounding countries in Central and South America [39]. In succeeding years, widespread infestations of this species were detected in numerous countries across the region (Fig. 1). Nowadays, *Aedes albopictus* is recorded in 13 countries in Central and South America and in 7 countries in the Caribbean (Fig. 1). Insufficient data are present documenting the invasion across this region to document the dispersal pathways at both global and continental spatial scales.

In Africa, as in Albania, Italy, France and Brazil, *Ae. albopictus* immatures were first detected inside used tyres in Cape Town, South Africa, in 1989 [62]. Imported tyres were transported via cargo ship from Tokyo, Japan, but established populations at that time or during the present day are not recorded in South Africa [62]. Following this detection in South Africa, *Ae. albopictus* was recorded in nine African countries, with the mode of dispersal into each country unknown at both global and continental spatial scales (Figs. 1, 2, Additional File 1: Table S1). Limited entomological records from Africa [63] suggest that the distribution of *Ae. albopictus* (and other mosquitoes) may be underestimated.

### Passive transportation by ground vehicles

This dispersal pathway is typically characterised by the unintentional transportation of container habitats with eggs or immatures and the passive dispersal of *Ae. albopictus* adults inside ground vehicles. This dispersal pathway is considered primarily to occur across national spatial scales, or across continental spatial scales in the case of countries with contiguous geography (such as in Europe) [6]. In the last two decades, this dispersal pathway has been recorded as a major contributor to first detections of *Ae. albopictus* into new countries in Europe (Fig. 3).

The used tyre trade transported via cargo ships was the main dispersal pathway at global and continental spatial scales for the introduction of *Ae. albopictus* into Europe [6] but ground vehicles are currently considered a major driving contributor to the rapid spread and dispersal of *Ae. albopictus* throughout Europe [5, 39, 64, 65]. Dispersal of adult *Ae. albopictus* by ground vehicles (e.g. trucks and private vehicles) from Italy is believed to have resulted in the dispersal of this species into Switzerland, Slovenia, San Marino, the Czech Republic, Croatia and Germany [16–21]. In Spain, dispersal of adult *Ae. albopictus* inside private vehicles across the country was directly observed in multiple instances [5], likely contributing to the rapid spread of *Ae. albopictus* throughout Spain since its first detection in 2004. Likewise, in France, this dispersal pathway was considered a key factor for *Ae. albopictus* range expansion throughout the country [66].

### Trade of lucky bamboo (*Dracaena* species)

The transportation of *Ae. albopictus* eggs on either plant stems or on gel or water used to transport lucky bamboo (*Dracaena* species) has been intercepted occasionally, with *Ae. albopictus* detected at lucky bamboo greenhouses in The Netherlands in 2005, 2010–2016 and in cargo shipments in Belgium in 2013, but populations did not become established in either country [22–24]. In California, USA, since 2000, multiple cargo shipments of lucky bamboo (*Dracaena* species) from Southeast Asia containing *Ae. albopictus* immatures were detected, with evidence of populations overwintering despite vector control efforts [67–69].

### Passive transportation by river boat

In Mali, *Ae. albopictus* immatures were found inside water-holding goods on small boats along the Niger River [70]. Given the affinity for *Ae. albopictus* dispersal via maritime transport, it is likely that transportation by river boat frequently occurs but is probably insufficiently surveyed.

### Infrequently recorded dispersal pathways

Dispersal pathways under this category are infrequently recorded in the published literature and have not been associated with the first introduction of *Ae. albopictus* to a new country. However, as these pathways have potential to introduce *Ae. albopictus* to new countries, it is worth documenting.

### Passive transportation by aircraft

The passive dispersal of *Ae. albopictus* aboard aircrafts has been confirmed infrequently in the published literature. Published records exist from The Netherlands [71], Australia [38] and New Zealand [72, 73]. The low incidence of *Ae. albopictus* records from this dispersal pathway may relate to aircraft disinsection, whereby aircrafts undergo spraying with pyrethroids, killing insects on board [74].

### Trade of plants or plant material (other than Lucky bamboo)

In The Netherlands, a single adult *Ae. albopictus* was captured at one of the largest flower auctions in Europe in 2017 [75]. Suppousedly, Lucky bamboo was absent from this auction, suggesting that *Ae. albopictus* was introduced via the trade of plants or plant material [6].

### Summary of dispersal pathways

Considering all known and suspected dispersal pathways for the introduction of *Ae. albopictus* in a new country, two main conclusions can be drawn: (i) at global and continental spatial scales, maritime sea transport was the main dispersal pathway for *Ae. albopictus* into new countries in the middle to late twentieth century, with ships carrying used tyres of particular importance during the 1980s and 1990s (Additional File 1: Table S1) and (ii) at continental and national spatial scales, the passive transportation of *Ae. albopictus* in ground vehicles and to a lesser extent the trade of used tyres and maritime sea transport appear to be the major drivers of *Ae. albopictus* dispersal into new countries, especially in Europe (Additional File 1: Table S1). Finally, it is worth noting that the dispersal pathways for the introduction and spread of *Ae. albopictus* in numerous countries remain unknown, especially from the 1990s onwards (Figs. 2, 3), where limited published information exists (i.e. countries in Central and South America, Africa, Australasia and Pacific Island nations).

A greater understanding of the dispersal pathways for *Ae. albopictus* introduction into countries is critical to vector surveillance strategies to detect and control future introductions. This next section will discuss techniques used to determine *Ae. albopictus* dispersal pathways focusing on their implications for vector surveillance programmes.

### Techniques for determining dispersal across different spatial scales

#### Vector surveillance

Understanding the dispersal pathways by which *Ae. albopictus* could invade new geographic areas allows for the development of more targeted vector surveillance and control programmes. Vector surveillance in select areas typically falls under the jurisdiction of local and regional governments, usually involving mosquito-control personnel from the health, quarantine and inspection sectors. Vector surveillance at PoE, usually managed by national government, is the front line of defence against the introduction of *Ae. albopictus* to new areas [12, 76]. Vector surveillance at PoE most commonly targets dispersal pathways at global and continental spatial scales. There are many different strategies and technologies required for successful vector surveillance programmes.

Entomological traps should be routinely deployed at high-risk PoE to monitor for potential *Ae. albopictus* incursions. Examples of traps that will sample for adult *Ae. albopictus* include: the BG-Sentinel (BGS) trap, CO_2_-baited mosquito light traps, the autocidal gravid ovitrap and the Gravid-*Aedes* Trap [13, 77]. Examples of traps that will sample adult larvae/eggs of *Ae. albopictus* include: oviposition traps and WHO standard tyre traps [13, 77]. Both the adult and larvae/egg traps listed can effectively detect *Ae. albopictus* [86–87]. However, notable limitations of these traps include overheads for equipment, large costs and time involved in servicing traps, and constraints of traps and technology to deliver information on the desired spatial scale required to inform officials about species invasions [79, 80].

Because there is no single effective tool for *Ae. albopictus* surveillance, the development of highly targeted tools for the detection of *Ae. albopictus*, is critical to determine their presence in new areas [15, 81]. The Male *Aedes* Sound Trap [82], a trap which exploits the female *Aedes* wing beat frequency to capture male *Aedes*, could also be appropriate for male *Ae. albopictus* surveillance [83, 84]. Additionally the use of adhesive tape for removing *Aedes* eggs from inside imported used tyres at PoE for rapid PCR-based identification holds promise as a low-cost method for sampling *Aedes* eggs directly from this high-risk cargo type [81].

Another recent development to improve the capacity to detect and monitor the spread of *Ae. albopictus* within a country is citizen science, where members of the public actively contribute to surveillance. Citizen science has the potential to be highly scalable with multiple collectors and the capacity to operate as ‘post-border’ (i.e. beyond the PoE) mosquito surveillance [85–87]. This could aid the objectives of vector surveillance, with multiple citizen science projects undertaken to collect *Ae. albopictus* data (Table 1). Citizen science projects and other communications from the public about nuisance mosquito biting resulted in the first detections of *Aedes* species in some countries. Citizen science first detected *Ae. albopictus* on the Spanish island of Ibiza [42], *Ae. japonicus* on the Spanish mainland [88], *Ae. camptorhynchus* in New Zealand [89] and *Ae. aegypti* and *Ae. koreicus* in Germany as well as monitoring the spread of *Ae. albopictus* and *Ae. japonicus* throughout this country [90, 91]. In response to the detection of these invasive *Aedes* species, traditional *Aedes* vector control techniques (e.g. widespread insecticide application and the deployment of mosquito traps) have been utilised for targeted vector control [89, 92]. However, citizen science for vector surveillance has notable limitations, including: sampling biases (citizens opt-in, possibly resulting in patchy geographic coverage), data quality (photos, need to be of high-quality for species identification by professionals) and the reliability of citizen scientists to make observations and collections has not been scientifically validated [87]. As such, citizen science may best serve as a complementary tool to existing entomological surveillance [93]. For example, when both citizen science and entomological surveillance were used in Spain, citizen science failed to detect *Ae. albopictus* in some areas which recorded positive collections in oviposition traps and vice versa [85].

**Table 1:** Examples of citizen science projects to collect data about *Aedes albopictus*. For a review comparing these projects see [94]

Purpose	Examples	Countries implemented in	Core tasks	Data submission (*A* = mobile app/website, *P* = postal)	References
Mosquito surveillance	Rapid surveillance for vector presence (RSVP)	South East Queensland, Australia	*Aedes* mosquito eggs are collected in citizen's yards from "Do-It-Yourself" oviposition traps (plastic bottle in black bucket with ovistrips). Egg strips are submitted for molecular identification	P	[95]
Mosquito surveillance & control	Citizen action through science	North East, USA	Citizens purchase, deploy and maintain Gravid *Aedes* Traps. Citizens are responsible for maintaining the traps (under supervision of community leaders and scientific advisors). Mosquito collections are undertaken by the citizen and delivered to a lab for identification	A & P	[96]
Mosquito surveillance and monitor adult populations in ‘real time’	Mosquito alert	Available in over 18 European countries	Geolocated pictures taken of mosquitoes. Associated ecological and sampling information added by citizen	A	[5, 85, 97]
	iMoustique	France		A	[92]
	iNaturalist mosquito projects	Various countries globally		A	[87]
Mosquito abundance and reported nuisance	Zanzamapp	Italy	Geolocated reports of nuisance biting caused by mosquitoes. Associated ecological and sampling information added by citizen	A	[98]

#### Genomic techniques

Capturing and monitoring *Ae. albopictus* specimens with vector surveillance, citizen science or other methods are increasingly being followed by the use of genomic techniques to trace the source of incursions at higher resolution [99]. Genotyping can be undertaken to identify the origin of insects by comparing the genotype of incursion samples to reference samples of known origin. Assignment of incursion samples to reference populations can then be initiated to identify the likely source population or location [99].

Knowledge about the source location and the dispersal pathways of invading *Ae. albopictus* is valuable in the strategic deployment of vector surveillance resources at source locations and PoE [100, 101]. Furthermore, new genomic techniques as used in population genomics [i.e. high resolution genetic markers, single nucleotide polymorphisms (SNPs)] allow mosquito dispersal pathways to be analysed more precisely [102, 103]. For example, to investigate the origin of *Ae. albopictus* invasions into Europe, at global and continental spatial scales, Sherpa et al. [104] sequenced individual *Ae. albopictus* collected in Europe and worldwide locations and showed that North and Central Italy were the major source of *Ae. albopictus* invasions throughout Europe. This finding corroborates the identified source countries of *Ae. albopictus* reported in the literature for Europe (Additional File 1: Table S1).

On the Australian mainland, genomic investigations examined the dispersal pathways of some *Ae. albopictus* detected at PoE (PoE in Brisbane, Darwin, Melbourne and Sydney) and traced the source locations to countries in East Asia, largely linked to maritime sea transport [38]. This information supports increased efforts in entomological surveillance in Australia and other countries for detecting this mosquito from these known source locations and likely dispersal pathways.

Where established populations of *Ae. albopictus* are detected beyond the PoE, genomics can investigate dispersal events which occur over more than one generation to estimate relatedness between kin [105–107]. Such approaches are scarce for *Ae. albopictus* [106] but their implementation could improve our understanding of dispersal to inform the spatial scale that vector surveillance and control efforts need to be deployed following incursions [108, 109]. In Australia, genomic techniques have been used to discover human-mediated dispersal of *Ae. albopictus* close kin tens of kilometres apart in the Torres Strait Islands [110], highlighting the difficulty of controlling this species in this region.

The use of genomic techniques relies on successfully capturing intact mosquito specimens (for high-quality DNA extraction), specialist laboratory facilities, molecular and bioinformatics expertise and funds to sequence samples. These factors alone may preclude some countries from embarking on using genomic techniques. However, the ever-decreasing costs of sequencing, availability of a hand-held portable sequencer [111] and a DNA sequence analysis mobile phone application [112] hold promise that in the future genomic techniques may be more accessible to a broader audience for improving vector surveillance.

## Conclusions

Over the past 80 years the global expansion of *Ae. albopictus* has been striking. Passive transportation by both maritime sea transport and ground vehicles has been the main dispersal pathways for *Ae. albopictus*, with ships and vehicles transporting used tyres of extremely high risk.

Preventing the establishment of *Ae. albopictus* requires significant ongoing investment in vector surveillance coupled with ongoing vector control at high-risk PoE and rapid responses to detections. This is likely to be exacerbated in the future with changes in global factors (e.g. land use, socioeconomic and climate change), which are likely to increase the rate of invasions and associated virus outbreaks vectored by *Ae. albopictus* [48, 113, 114].

For countries with contiguous geography, the likelihood of *Ae. albopictus* dispersal appears heightened, evidenced by the rapid spread and dispersal of *Ae. albopictus* throughout Europe via ground vehicles [5, 39, 64, 65]. For countries with non-contiguous geography, particular focus of surveillance efforts should be directed to high-risk dispersal pathways, such as the trade of used tyres and the passive transportation by maritime sea transport. Modelling estimates that *Ae. albopictus* will be reported in 197 countries by 2080 [48], a rapid increase from the 86 countries where *Ae. albopictus* was reported between 1940–2020. Focus for countries where *Ae. albopictus* is not yet established should be directed to improving the capacity to detect this species at and beyond the PoE by integrating relevant advances in vector surveillance and genomic techniques.

Successful strategies require improvements in highly specific traps for capturing *Ae. albopictus*. Deployment of low-power, cost-effective traps could greatly expand vector surveillance around the globe. Integration with existing citizen science systems holds promise in providing platforms for upscaling and improving vector surveillance, potentially at lower cost than governments deploying entomological traps [85, 87]. However, gaps in our understanding of dispersal pathways of *Ae. albopictus* still exist including the origins of invading individuals utilising these pathways. Genomic techniques can answer these questions and uptake in the future should increase as sequencing costs decrease and the tools to interpret the results become more user-friendly.

Finally, global collaboration is required to seamlessly share data between countries (i.e. cloud-based online systems) about *Ae. albopictus* detections in new areas. Such data-sharing alone has great potential to enhance global *Ae. albopictus* surveillance and control.

## Supplementary Information

**Additional file 1: Table S1.** Overview of *Aedes albopictus* known and suspected dispersal pathways across different spatial scales for the period 1940–2020. Publications reporting the first introduction of *Ae. albopictus *in a new country were selected. Colours indicate the spatial scale at which this dispersal event was recorded (gold = global; green = continental; grey = unknown). “Unknown” dispersal pathway or spatial scale is defined from published scientific articles with insufficient evidence to prove or suspect otherwise. “Suspected” dispersal pathway in this context refers to evidence from publications which indicates that this is the most likely dispersal pathway or spatial scale (e.g. *Ae. albopictus *immatures found in used tyres transported from Japan to the USA). "Unknown" trap type is defined as not documented in publications. *Establishment status was determined from published scientific articles, reports, agencies and organisations (i.e. ECDC and CDC) documenting persistence. A = adults, L = larvae, E = eggs. HB = human bait, LD = larval dipper, DIT = dry ice, T = tyre trap, O = oviposition trap, BGS = Biogents sentinel trap, EVS = Encephalitis Virus Surveillance trap, CDC = Centers for Disease Control light trap, NT = net trap, E = emergence trap, A = aspirator used to collect adult mosquitoes.

## Data Availability

Not applicable.

## Abbreviations

PoE: Points of entry
CDC: Centers for Disease Control and Prevention
ECDC: European Centre for Disease Prevention and Control
