# Supplementary material for: A literature review of dispersal pathways of Aedes albopictus across different spatial scales: implications for vector surveillance
Source: Parasit Vectors. 2022 Aug 27;15:303. doi: 10.1186/s13071-022-05413-5 (PMC9420301; doi:10.1186/s13071-022-05413-5)
Supplement: Supplementary file 1 — Additional file 1: Table S1. Overview of Aedes albopictus known and suspected dispersal pathways across different spatial scales for the period 1940–2020. Publications reporting the first introduction of Ae. albopictus in a new country were selected. Colours indicate the spatial scale at which this dispersal event was recorded (gold = global; green = continental; grey = unknown). “Unknown” dispersal pathway or spatial scale is defined from published scientific articles with insufficient evidence to prove or suspect otherwise. “Suspected” dispersal pathway in this context refers to evidence from publications which indicates that this is the most likely dispersal pathway or spatial scale (e.g. Ae. albopictus immatures found in used tyres transported from Japan to the USA). "Unknown" trap type is defined as not documented in publications. *Establishment status was determined from published scientific articles, reports, agencies and organisations (i.e. ECDC and CDC) documenting persistence. A = adults, L = larvae, E = eggs. HB = human bait, LD = larval dipper, DIT = dry ice, T = tyre trap, O = oviposition trap, BGS = Biogents sentinel trap, EVS = Encephalitis Virus Surveillance trap, CDC = Centers for Disease Control light trap, NT = net trap, E = emergence trap, A = aspirator used to collect adult mosquitoes. [file 13071_2022_5413_MOESM1_ESM.docx]

**Supplementary Information**

**Additional File 1: Table S1:** Overview of *Aedes albopictus* known and suspected dispersal pathways across different spatial scales for the period 1940*–*2020. Publications reporting the first introduction of *Ae. albopictus* in a new country were selected. Colours indicate the spatial scale which this dispersal event was recorded (Gold = Global, Green = Continental and Grey = Unknown). “Unknown” dispersal pathway or spatial scale is defined from published scientific articles with insufficient evidence to prove or suspect otherwise. “Suspected” dispersal pathway in this context refers to evidence from publications which incriminates that this is the most likely dispersal pathway or spatial scale (e.g. *Ae. albopictus* immatures found in used tyres transported from Japan to the USA). "Unknown" trap type is defined as not documented in publications. * Establishment status was determined from published scientific articles, reports, agencies and organisations (i.e. ECDC and CDC) documenting persistence. A = adults, L = larvae, E = eggs. HB = human bait, LD = larval dipper, DIT = dry ice, T = tyre trap, O = oviposition trap, BGS = Biogents sentinel trap, EVS = Encephalitis Virus Surveillance trap, CDC = Centre for Disease Control light trap, NT = Net trap, E = Emergence trap, A = Aspirator used to collect adult mosquitoes.

| **Dispersal pathway** | **Spatial scale** | **Time period** | **Year first detected** | **Recipient country (Region)** | **Donor country (Region)** | **Lifestage detected** | **Trap used** | **Detected at PoE?** | **Established?*** | **References** |
| --- | --- | --- | --- | --- | --- | --- | --- | --- | --- | --- |
| Used tyres | Global | 1940-1949 | 1946 | USA (North America) | Philippines (Asia) | A + L | HB + LD | Y | Y | [45] |
| Used tyres | Global | 1980-1989 | 1986 | Brazil (Central & South America) | (Asia) | A | Unknown | N | Y | [59] |
| Used tyres | Global | 1980-1989 | 1989 | Albania (Europe) | China (Asia) | A + L | HB + LD | N | Y | [52] |
| Used tyres | Global | 1980-1989 | 1989 | South Africa (Africa) | Japan (Asia) | L | LD | Y | N | [62] |
| Used tyres | Global | 1990-1999 | 1990 | Italy (Europe) | USA (North America) | A + L | HB + LD | N | Y | [54,55] |
| Used tyres | Global | 1990-1999 | 1993 | New Zealand (Australasia & Pacific Islands) | Japan (Asia) | A + L | LD | Y | N | [115] |
| Used tyres | Global | 1990-1999 | 1999 | France (Europe) | Japan (Asia) or USA (North America) | A + L | HB + LD + DIT | N | Y | [53] |
| Lucky bamboo | Global | 2000-2009 | 2005 | Netherlands (Europe) | China (Asia) | A | HB | N | N | [22] |
| Used tyres | Global | 2000-2009 | 2000 | Belgium (Europe) | Japan (Asia) or USA (North America) | L | LD | N | N | [116] |
| Maritime sea transport | Continental | 1940-1949 | 1944 | Guam (Australasia & Pacific Islands) | (Asia) | A + L | HB + LD | N | Y | [26] |
| Unknown | Continental | 1960-1969 | 1962 | Indonesia (Australasia & Pacific Islands) | (Australasia & Pacific Islands) | Unknown | Unknown | N | Y | [117] |
| Maritime sea transport | Continental | 1970-1979 | 1972 | Papua New Guinea (Australasia & Pacific Islands) | (Australasia & Pacific Islands) | Unknown | Unknown | N | Y | [27] |
| Maritime sea transport | Continental | 1970-1979 | 1978 | Solomon Islands (Australasia & Pacific Islands) | PNG (Australasia & Pacific Islands) | A + L | LD + HB | N | Y | [28] |
| Unknown | Continental | 1980-1989 | 1988 | Mexico (North America) | USA (Central & South America) | L | LD | N | Y | [118] |
| Unknown | Continental | 1980-1989 | 1989 | Fiji (Australasia & Pacific Islands) | PNG (Australasia & Pacific Islands) | A | Unknown | Y | Y | [29] |
| Ground vehicles | Continental | 1990-1999 | 1991 | Palestine (Middle East) | Israel (Middle East) | A + L | LD + HB | N | Y | [119] |
| Maritime sea transport | Continental | 1990-1999 | 1995 | Cuba (Central & South America) | Unknown | L | LD | N | Y | [120] |
| Maritime sea transport | Continental | 1990-1999 | 1997 | Cayman Islands (Central & South America) | Florida (Central & South America) | L | O | N | Y | [121] |
| Unknown | Continental | 1990-1999 | 1993 | Dominican Republic (Central & South America) | Unknown | L | T | N | Y | [122] |
| Unknown | Continental | 1990-1999 | 1998 | Colombia (Central & South America) | Brazil (Central & South America) | A | Unknown | N | Y | [123] |
| Unknown | Continental | 1990-1999 | 1998 | Argentina (Central & South America) | Brazil (Central & South America) | A + L | LD + E | N | Y | [124] |
| Unknown | Continental | 1990-1999 | 1990s | Honduras (Central & South America) | (Central & South America) | L | Unknown | N | Y | [125] |
| Ground vehicles | Continental | 2000-2009 | 2001 | Montenegro (Europe) | Unknown | A | Unknown | N | Y | [17] |
| Ground vehicles | Continental | 2000-2009 | 2003 | Greece (Europe) | Italy (Europe) | A | HB | N | Y | [40] |
| Ground vehicles | Continental | 2000-2009 | 2003 | Switzerland (Europe) | Italy (Europe) | A + E | O | N | N | [16] |
| Ground vehicles | Continental | 2000-2009 | 2004 | Croatia (Europe) | (Europe) | L | LD | N | Y | [20] |
| Ground vehicles | Continental | 2000-2009 | 2005 | Bosnia & Herzegovina (Europe) | (Europe) | A | Unknown | N | Y | [17] |
| Ground vehicles | Continental | 2000-2009 | 2006 | Monaco (Europe) | France or Italy (Europe) | A | Unknown | N | Y | [18,126] |
| Ground vehicles | Continental | 2000-2009 | 2007 | Germany (Europe) | Northern Italy (Europe) | L | LD | N | N | [21] |
| Ground vehicles | Continental | 2000-2009 | 2007 | Slovenia (Europe) | Italy (Europe) | NA | Unknown | N | Y | [17] |
| Ground vehicles | Continental | 2000-2009 | 2007 | San Marino (Europe) | Italy (Europe) | NA | Unknown | N | Y | [18,126] |
| Maritime sea transport | Continental | 2000-2009 | 2002 | Corsica, France (Europe) | Unknown | Unknown | Unknown | N | Y | [39] |
| Maritime sea transport | Continental | 2000-2009 | 2003 | Greek Islands, Greece (Europe) | Unknown | Unknown | Unknown | N | Y | [40] |
| Maritime sea transport | Continental | 2000-2009 | 2004 | Torres Strait, Australia (Australasia & Pacific Islands) | Indonesia (Australasia & Pacific Islands) | L | BGS + LD + HB | N | Y | [31] |
| Maritime sea transport | Continental | 2000-2009 | 2009 | Malta (Europe) | Italy (Europe) | A + L | HB + LT + LD | N | Y | [41] |
| Unknown | Continental | 2000-2009 | 2001 | Canada (North America) | North America | A | Unknown | N | Y | [127] |
| Unknown | Continental | 2000-2009 | 2003 | Nicaragua (Central & South America) | (Central & South America) | L | LD | N | Y | [128] |
| Unknown | Continental | 2000-2009 | 2004 | Spain (Europe) | (Europe) | A + L | HB + LD | N | Y | [129] |
| Unknown | Continental | 2000-2009 | 2004 | Panama (Central & South America) | (Central & South America) | A + L + E | HB + O | N | Y | [130] |
| Unknown | Continental | 2000-2009 | 2009 | Costa Rica (Central & South America) | (Central & South America) | A | HB | N | Y | [131] |
| Unknown | Continental | 2000-2009 | 2009 | Central African Republic (Africa) | Cameroon (Africa) | A + L | LD + HB | N | Y | [132] |
| Ground vehicles | Continental | 2010-2019 | 2011 | Russia (Europe) | (Europe) | A + L | HB + LD | N | Y | [133] |
| Ground vehicles | Continental | 2010-2019 | 2011 | Turkey (Europe) | (Europe) | L | O | N | Y | [134] |
| Ground vehicles | Continental | 2010-2019 | 2012 | Romania (Europe) | (Europe) | L | LD | N | Y | [135,136] |
| Ground vehicles | Continental | 2010-2019 | 2012 | Slovakia (Europe) | (Europe) | A | CDC | N | Unknown | [137] |
| Ground vehicles | Continental | 2010-2019 | 2012 | Austria (Europe) | (Europe) | A + L | O | N | N | [138] |
| Ground vehicles | Continental | 2010-2019 | 2012 | Czech Republic (Europe) | Italy (Europe) | L | LD | N | Y | [19] |
| Ground vehicles | Continental | 2010-2019 | 2016 | England (Europe) | (Europe) | A + L + E | O | Y | N | [139] |
| Ground vehicles | Continental | 2010-2019 | 2016 | Macedonia (Europe) | (Europe) | L | O | N | Y | [140] |
| Ground vehicles | Continental | 2010-2019 | 2016 | Armenia (Europe) | (Europe) | A + L | LD + EVS + CDC + BGS | N | Y | [141] |
| Maritime sea transport | Continental | 2010-2019 | 2014 | Ibiza, Spain (Europe) | Europe | A | HB + CDC + O | N | Y | [42] |
| Maritime sea transport | Continental | 2010-2019 | 2016 | Tyrrhenian islands, Italy (Europe) | Italy (Europe) | L | LD + O | N | Y | [43] |
| Unknown | Continental | 2010-2019 | 2010 | Haiti (Central & South America) | (Central & South America) | L | LD | N | Y | [142] |
| Unknown | Continental | 2010-2019 | 2011 | Bulgaria (Europe) | (Europe) | Unknown | Unknown | Unknown | Y | [134] |
| Unknown | Continental | 2010-2019 | 2012 | Hungary (Europe) | (Europe) | Unknown | Unknown | Unknown | Y | [143] |
| Unknown | Continental | 2010-2019 | 2014 | Georgia (Europe) | (Europe) | A | CDC | N | Y | [144] |
| Unknown | Continental | 2010-2019 | 2016 | Democratic Republic of Congo (Africa) | (Africa) | A + L | LD + HB | N | Y | [145] |
| Unknown | Continental | 2010-2019 | 2016 | Sao Tome and Principe (Africa) | Cameroon (Africa) | A + L | HB + NT + LD | N | Y | [146] |
| Unknown | Continental | 2010-2019 | 2017 | Gibraltar (Europe) | (Europe) | Unknown | Unknown | Unknown | Y | [147] |
| Used tyres | Continental | 2010-2019 | 2017 | Portugal (Europe) | (Europe) | A + L | CDC + BGS + O | Y | Y | [148] |
| Unknown | Unknown | 1990-1999 | 1991 | Nigeria (Africa) | Unknown | E | O | N | Y | [149] |
| Unknown | Unknown | 1990-1999 | 1995 | El Salvador (Central & South America) | Unknown | L | Unknown | N | Y | [125] |
| Unknown | Unknown | 1990-1999 | 1995 | Guatemala (Central & South America) | Unknown | L | LD | N | Y | [150] |
| Unknown | Unknown | 1990-1999 | 1990s | Paraguay (Central & South America) | Unknown | Unknown | Unknown | Unknown | Unknown | [125] |
| Unknown | Unknown | 2000-2009 | 2000 | Cameroon (Africa) | Unknown | A + L | LD + HB | N | Y | [151] |
| Unknown | Unknown | 2000-2009 | 2001 | Equatorial Guinea (Africa) | Unknown | A + L | LD + CDC + HB | N | Y | [152] |
| Unknown | Unknown | 2000-2009 | 2002 | Trinidad & Tobago (Central & South America) | Unknown | A + L | O + HB | N | N | [153] |
| Unknown | Unknown | 2000-2009 | 2002 | Israel (Middle East) | Unknown | L | T | Y | Y | [154] |
| Unknown | Unknown | 2000-2009 | 2003 | Lebanon (Middle East) | Unknown | L | LD | N | Y | [155] |
| Unknown | Unknown | 2000-2009 | 2003 | Uruguay (Central & South America) | Unknown | Unknown | Unknown | Unknown | Unknown | [156] |
| Unknown | Unknown | 2000-2009 | 2005 | Syria (Middle East) | Unknown | A | HB | N | Y | [155] |
| Unknown | Unknown | 2000-2009 | 2006 | Gabon (Africa) | Unknown | A + L | LD + CDC + HB | N | Y | [157] |
| Unknown | Unknown | 2000-2009 | 2009 | Belize (Central & South America) | Unknown | A | HB | N | Y | [158] |
| Unknown | Unknown | 2000-2009 | 2009 | Iran (Middle East) | Unknown | A + L | LD + HB + CDC | N | N | [159] |
| Unknown | Unknown | 2000-2009 | 2009 | Venezuela (Central & South America) | Unknown | L | LD | N | Y | [160] |
| River boats | Unknown | 2010-2019 | 2012 | Mali (Africa) | Unknown | L | HB + LD | N | Y | [70] |
| Unknown | Unknown | 2010-2019 | 2010 | Algeria (Africa) | Unknown | A | Unknown | N | Y | [161] |
| Unknown | Unknown | 2010-2019 | 2012 | Tonga (Australasia & Pacific Islands) | Unknown | L | LD | N | Y | [30] |
| Unknown | Unknown | 2010-2019 | 2015 | Mozambique (Africa) | Unknown | A | HB | N | Unknown | [162] |
| Unknown | Unknown | 2010-2019 | 2015 | Morocco (Africa) | Unknown | L | LD | N | Y | [163] |
| Unknown | Unknown | 2010-2019 | 2016 | Jordan (Middle East) | (Asia) | A + L | LD + HB | N | Y | [164] |
| Unknown | Unknown | 2010-2019 | 2017 | Ecuador (Central & South America) | Unknown | A | BGS | N | Unknown | [165] |
| Unknown | Unknown | 2010-2019 | 2018 | Tunisia (Africa) | Unknown | A + L | BGS + O + CS | N | Unknown | [166] |
| Unknown | Unknown | 2010-2019 | 2018 | Jamaica (Central & South America) | Unknown | A | BGS + CDC | N | Unknown | [167] |
| Unknown | Unknown | 2010-2019 | 2010s | Kiribati (Australasia & Pacific Islands) | Unknown | Unknown | Unknown | Unknown | Unknown | [13] |
| Unknown | Unknown | 2010-2019 | 2010s | Marshall Islands (Australasia & Pacific Islands) | Unknown | Unknown | Unknown | Unknown | Unknown | [13] |
| Unknown | Unknown | 2010-2019 | 2010s | Northern Mariana Islands (Australasia & Pacific Islands) | Unknown | Unknown | Unknown | Unknown | Unknown | [13] |
| Unknown | Unknown | 2010-2019 | 2010s | Palau (Australasia & Pacific Islands) | Unknown | Unknown | Unknown | Unknown | Unknown | [13] |
| Unknown | Unknown | 2010-2019 | 2010s | Samoa (Australasia & Pacific Islands) | Unknown | Unknown | Unknown | Unknown | Unknown | [13] |
| Unknown | Unknown | 2010-2019 | 2010s | Vanuatu (Australasia & Pacific Islands) | Unknown | Unknown | Unknown | Unknown | Unknown | [30] |
